# Demographic composition of National Institutes of Health Clinical and Translational Science Awards (CTSA) Program principal investigators, scholars, and trainees

**DOI:** 10.1017/cts.2022.491

**Published:** 2022-11-09

**Authors:** Mercedes Rubio, Heather L. Baker, Jamie Mihoko Doyle

**Affiliations:** 1 National Institute of General Medical Sciences, Bethesda, MD, USA; 2 National Center for Advancing Translational Sciences, Bethesda, MD, USA

**Keywords:** CTSA, CTSA hub and training components principal investigators, CTSA KL2 scholars, CTSA TL1 trainees, race and ethnicity, gender, diversity, equity and inclusion

## Abstract

Little has been published on the demographic composition of the clinical and translational science research workforce within the Clinical and Translational Science Awards (CTSA) Program despite the well-documented need for greater diversity in the biomedical research workforce. Analyses of workforce demographic reveal that women and members of underrepresented groups remain persistently underrepresented in the CTSA hub and training components principal investigators. In contrast, in the CTSA Program career development and training programs, females have greater representation as participants, and non-Whites were better represented in training programs.

## Introduction

The Clinical and Translational Science Awards (CTSA) Program was established by the National Institutes of Health (NIH) to develop and implement innovative solutions that improve the efficiency, quality, and impact of the process for turning observations in the laboratory, clinic, and community into interventions that improve the health of individuals and communities. The conceptualization of the CTSA Program called for a national network of medical research institutions (referred to as “hubs”) to integrate intellectual and physical resources to enhance their abilities to conduct original clinical and translational science (CTS) research [1]. Indeed, the CTSA Program has transformed and increased the academic standing of the CTS enterprise [2]. This success is due in part to an overall structure that promotes and encourages collaboration and the sharing of best practices – both outside and across this national network of CTSA Program institutions. In Fiscal Year (FY) 2021, NCATS invested approximately over $550 million dollars and supported 61 CTSA Programs which serve as the focal points for CTS [3].

The long-term sustainability of this investment includes a complementary commitment to training the next generation of CTS researchers. The development and retention of a well-trained and diverse CTS workforce, who would lead research to improve human health, have been woven into the CTSAs’ fabric since its inception [4]. Until recently, each CTSA application (funded under a U54 cooperative agreement mechanism) consisted of 2–3 parts – the UL1, KL2, and TL1 – that were submitted as a single application and separated at the time of award. The primary infrastructure component (i.e., UL1) provides the broad, trans-institutional structure that promotes CTS research at the institution and across partners and collaborators. The Institutional Mentored Career Development Award (i.e., KL2) is a required component and supports early-career postdoctoral researchers who advance CTS. The National Research Service Award (NRSA) (i.e., TL1) is optional and supports predoctoral, postdoctoral, and short-term appointment trainees. The CTSA Program is recognized for its robust and successful career development and research training programs [2]. Despite documented successes, a 2013 Institute of Medicine report recommended increasing the ethnic/racial diversity of mentors, scholars, and trainees within the CTSA Program [5].

The NIH recognizes the importance of and the need for greater diversity in the biomedical research workforce from multiple perspectives such as, but not limited to, race, ethnicity, and gender [6]. Despite NIH’s longstanding efforts to increase diversity in the biomedical workforce – from undergraduates to established investigators — a persistent racial gap remains [7]. In 2017, the NIH Advisory Committee to the Director Working Group on Diversity considered funding disparities as a serious issue that required attention. The recommendation called for a more deliberate look at institutional systems and processes to address systems level and culture change [7]. Systems level and culture change both impact not only the timely transition of individuals to career independence, but also the demographic composition of leadership positions at academic institutions [8].

A rich body of research documents the importance of attracting, developing, and supporting the best scientists from all groups, at all training and career stages levels (including leadership), and how diversity contributes to a strong, robust biomedical research enterprise [9]. Diverse groups boost towards new ideas, methodologies, approaches, and research questions than homogenous groups [10]. NCATS’ Strategic Plan emphasizes the need for a robust translational science workforce [11]. Strategic Goal #3, for example, recognizes the importance of a diverse workforce and its contribution to advancing CTS [11]. Specifically, this strategic goal recognizes that multiple perspectives bring creativity and ingenuity to complex translational problems, thus improving health through smarter science [11].

To our knowledge, the demographic characteristics of the CTSA hub and training component principal investigators (PIs) (i.e., recipients of UL1, KL2, and TL1 awards; multi-PIs included), the KL2 scholar, and TL1 trainee populations have never been published [12]. Given the maturation of the CTSA Program, NCATS celebrating its 10^th^ anniversary, and NCATS’ commitment to Diversity, Equity, and Inclusion (DEI), this report is timely. The purpose of this report is to describe the diversity of CTSA hub and training components PIs, KL2 scholars, and TL1 trainees. We use the Notice of NIH’s Interest in Diversity [13] as a framework for defining diversity, which includes gender and race/ethnicity. Unfortunately, individuals from disadvantaged backgrounds are not examined. These data became available in FY 2019 and there are too few individuals with disabilities among CTSA hub and training component PIs, trainees, and scholars to report their representation without identifying them.

## Methods

Data for this report come from restricted-use administrative data (i.e., IMPAC II system) obtained from the NIH Office of Extramural Research. All CTSA UL1, KL2, and TL1 PIs, including multi-PIs, from FY 2011–2020 are included as well as KL2 scholars and TL1 trainees appointed from FY 2011–2020. Demographic data are self-reported as entered on each individual’s electronic Research Administration (eRA) Commons^1^ profile. Racial/ethnic categories are based on the Office of Management and Budget Statistical Directive 15 (OMB 15) classifications, which are required of all federal agencies collecting administrative data on race and ethnicity. Racial categories include African American/Black; American Indian or Alaskan Native; Asian; Native Hawaiian or Other Pacific Islander; “Other” race; and White. Individuals are permitted to self-identify with more than one race on their eRA Commons profile, and they are considered underrepresented minorities in this report regardless of the specific races or ethnicity that were specified. In addition to these racial categories, individuals can also identify as Hispanic or non-Hispanic as their ethnicity. Those who self-identified as “White” and “non-Hispanic” are coded as “non-Hispanic White.” Individuals can self-identify as male or female for their gender. There is no requirement for individuals to report these demographic characteristics.

Data aggregation across multiple years for specific FYs was used to ensure adequate suppression of cell sizes (N > 11) to prevent the identification of any PI, scholar, or trainee based on their demographic characteristics. For percentage distributions by gender (% female), FY 2011 through 2015 were combined to ensure sufficient cell suppression. The data for FY 2016 to FY 2020 are presented by year. Similarly, for percentage distributions by race/ethnicity (% non-Hispanic White), data from 2011 through 2015 were also combined. Comparisons of CTSA PIs, trainees, and scholars to others were made by using publicly available existing data.

### Principal Investigators-Comparison Groups

The NIH Data Book, descriptor, was the source of percentages of NIH Research Project Grant (RPG) and NIH Center Grant PI who were female and who were White (RPG only) [14, 15]. RPGs were defined as grants from the following activity codes: Those beginning with R, P, M, S, K, U (excluding UC6) in addition to DP1, DP2, DP3, DP4, DP5, D42, and G12. NIH Center Grants were defined by the following activity codes: G12, M01, P20, P30, P40, P41, P42, P50, P51, P60, PL1, U30, U40, U41, U42, U50, U51, U54, UL1, and R07 activity codes. It is important to note that data from the NIH Data Book for the percentage of White RPG PIs are not directly comparable to data from NCATS because Hispanics who self-identify as White are included in the NIH Data Book, but not for the percentage of non-Hispanic White PIs for NCATS.

Data on medical school faculty and deans come from the Association of American Medical Colleges (AAMC) [16]. It is important to note that gender data on medical school deans were not available for 2019 or by race/ethnicity for all years considered for this report, and that the AAMC data are based on the academic year whereas NIH data are based on FY.

### Trainees and Scholars Comparison Groups

CTSA KL2 scholars and TL1 trainees were compared with all NIH Mentored Research Career awardees and Training Grant trainees, respectively, using data from the NIH Data Book and IMPAC II. Mentored Research Career Awards included the following activity codes: K01, K07, K08, K22, K23, K25, K99, KL1, and KL2. Training Grants included the following activity codes: T32 and T35. Data on all NIH Mentored Research Career Awardees and Training Grant trainees were taken from the NIH Data Book, but information about gender was not available for FY 2019 and FY 2020. Data by race/ethnicity were not available for Mentored Research Career Awards and Institutional Research Training Awards overall by FY in the NIH Data Book. These data were requested from the NIH Office of Extramural Research and drawn from IMPAC II.

## Results

### Gender Distribution of CTSA Program Hub Leadership PIs

Table 1 displays the gender distribution of CTSA Program PIs as percent female. In the early years of the CTSA Program (FY 2011–2015), 18% of CTSA Program PIs were female. The data show an upward trend in the female representation between FY 2016 and FY 2018, from 28% to 32%, respectively. In FY 2019 female representation was 32%, and in FY 2020, the data show an upward trend to 34%. During the same time frame, there was a slight upward trend in female representation as PIs on NIH RPGs from 31% (FY 2011–2015) to 34% (FY 2019); NIH Center Grants from 21% (FY 2011–2015) to 26% (FY 2019); and in medical school deans from 16% (2016 academic year) to 18% (2018 academic year).

**Table 1.** Percentage distribution (%) and count (N) of female clinical and translational science awards (CTSA) principal investigators (PIs) (combined activity codes: UL1, KL2, and TL1) as compared to national institutes of health (NIH) research project grant (RPG), NIH center grants, and medical school deans by fiscal year (FY)

*% Female* (Total N)	FY 2011–15	FY 2016	FY 2017	FY 2018	FY 2019	FY 2020
CTSA Leadership*	18% (355)	28% (127)	29% (162)	32% (201)	32% (216)	34% (218)
NIH RPG Funding*	31%†	32%	32%	33%	34%	NA
NIH Center Grants*^^^	21%	23%	25%	26%	26%	NA
Medical School Deans^#^	NA	16%	17%	18%	NA	NA

* These data come from NIH IMPAC II database. The CTSA leadership is operationalized as the CTSA Hub and training components principal investigators, multi-PI included.

† This percentage represents the average of FY 2011–2015.

^ Research Center Grants are defined as G12, M01, P20, P30, P40, P41, P42, P50, P51, P60, PL1, U30, U40, U41, U42, U50, U51, U54, UL1, and R07 activity codes. Not all of these activities may be in use by NIH every year. The race and ethnicity of PIs of Center Grants are not available.

# The data come from the Association of American Medical Colleges (AAMC). These data are based on the academic calendar and not on the FY. Source: https://www.aamc.org/data-reports/faculty-institutions/report/state-women-academic-medicine

NA: not available.

### Racial/Ethnic Distribution of CTSA Program Hub Leadership PIs

Table 2 displays the percent of CTSA Program PI leadership who are non-Hispanic White, where data are available, we compare with non-Hispanic White representation in the NIH RPG funding portfolio and among medical school faculty. Consistently, between FY 2011 and FY 2020, roughly 80% or more of the CTSA Program PI leadership has been non-Hispanic White. In contrast, the percent of non-Hispanic Whites in the NIH RPG portfolio has remained at an average of 74% between FY 2016 and FY 2019. Between the 2011 and 2019 academic years, medical school faculty slightly trended towards more diverse. Between the 2011–2015 academic years, 67% of the medical school faculty members were non-Hispanic White. By the 2019 academic year, the percent of non-Hispanic White was 63%. This is a 4% absolute difference. These data show that the CTSA Program PI leadership is less diverse relative to those in the NIH RPG funding portfolio and among medical school faculty.

**Table 2.** Percentage (%) and count (N) of non-Hispanic white CTSA principal investigators (PIs) represented in hub leadership (combined activity codes: UL1, KL2, and TL1), by fiscal year (FY)

*% White* (Total N)	FY 2011–15	FY 2016	FY 2017	FY 2018	FY 2019	FY 2020
CTSA Leadership*	87% (355)	86% (127)	82% (162)	83% (201)	83% (216)	79% (218)
NIH RPG Funding*	NA	75%	75%	74%	73%	73%
Medical School Faculty^@^	67%	65%	64%	64%	63%	NA

* These data come from NIH IMPAC II data base. The CTSA leadership is operationalized as the CTSA Hub and training components principal investigators, multi-PI included.

@ Medical School Faculty is defined as individuals who are a full-time faculty at US medical schools. These data are based on the academic calendar and not the FY. Source: https://www.aamc.org/data-reports/faculty-institutions/interactive-data/us-medical-school-faculty-trends-percentages

NA: not available.

Along with exploring the demographic characteristics of the CTSA Program leadership, we report the demographic composition of the CTSA Program scholars and trainees between FY 2011 and FY 2020 (Tables 3 and 4). We compare the scholar and trainee populations to the NIH Mentored Research Career Awardees (inclusive of the K Institutional awards) and Institutional Training Grants.

**Table 3.** Percentage (%) and count (N) of female KL2 scholars and TL1 trainees, by fiscal year (FY)

Activity	Gender	FY11	FY12	FY13	FY14	FY15	FY16	FY17	FY18	FY19	FY20
KL2*	Total (N)	437	473	441	440	452	410	393	368	419	404
CTSA KL2 Scholars*	% Female	54%	55%	57%	53%	56%	57%	60%	58%	56%	57%
Institutional Ks*^§^	% Female	47%	47%	47%	48%	52%	51%	51%	52%	NA	NA
TL1*^¶^	Total (N)	370	348	361	344	408	499	537	566	592	532
CTSA TL1 Trainees	% Female	56%	58%	54%	54%	53%	54%	54%	59%	58%	58%
Institutional Ts*	% Female	53%	54%	55%	54%	52%	55%	55%	53%	NA	NA

* These data come from NIH IMPAC II data base.

§ Mentored Research Career Awards include activity codes: K01, K07, K22, K23, K99, and KL2; Total (N) not available.

¶ Training Grants: Activity codes T32 and T35; Total (N) not available.

NA: not available.

**Table 4.** Count (N) of institutional KL2/K scholars and institutional research training grants TL1/T trainees and percentage (%) of KL2 scholars and TL1 trainees, by race/ethnicity category and fiscal year (FY)

Activity	Race/Ethnicity Category	FY11	FY12	FY13	FY14	FY15	FY16	FY17	FY18	FY19	FY20
KL2*	Total (N)	437	473	441	440	452	410	393	368	419	404
	% Non-Hispanic Asian	21%	22%	22%	20%	20%	20%	20%	18%	18%	22%
	% Non-Hispanic White	63%	62%	60%	61%	59%	60%	58%	56%	55%	53%
	% Underrepresented Groups^∞^	12%	12%	13%	13%	15%	15%	18%	21%	22%	22%
Institutional Ks*ˠ	Total (N)	3353	3278	3157	3160	3147	3182	3286	3517	3696	3932
	% Non-Hispanic Asian	20%	21%	21%	21%	21%	20%	21%	21%	21%	20%
	% Non-Hispanic White	58%	57%	58%	60%	59%	59%	58%	58%	57%	56%
	% Underrepresented Groups	17%	17%	17%	15%	15%	16%	16%	16%	17%	18%
TL1*	Total (N)	370	348	361	344	408	499	537	566	592	532
	% Non-Hispanic Asian	21%	21%	23%	21%	21%	20%	22%	15%	15%	15%
	% Non-Hispanic White	56%	53%	56%	52%	54%	54%	52%	59%	56%	57%
	% Underrepresented Groups	17%	19%	16%	22%	18%	20%	20%	21%	24%	24%
Institutional Ts*ⸯ	Total (N)	13,860	13,308	12,440	12,261	12,088	12,273	12,459	12,418	12,081	12,093
	% Non-Hispanic Asian	16%	16%	16%	16%	16%	16%	16%	17%	17%	17%
	% Non-Hispanic White	62%	61%	60%	60%	60%	58%	57%	57%	57%	56%
	% Underrepresented Groups	19%	19%	20%	20%	20%	21%	22%	22%	23%	24%

* These data come from NIH IMPAC II data base.

∞ The following racial and ethnic groups have been shown to be underrepresented in biomedical research: Blacks or African Americans, Hispanics, or Latinos, American Indians or Alaska Natives, Native Hawaiians, and other Pacific Islanders. See NOT-OD-20-031.

ˠ Mentored Research Career Awards include activity codes: K01, K07, K08, K22, K23, K25, K99, KL1, and KL2.

ⸯ Training Grants: Activity codes T32 and T35.

### Gender Distribution of CTSA Program Scholars and Trainees

Unlike the representation of women among the CTSA Program hub PIs, females are better represented in the KL2 and TL1 population (Table 3). The data show that females consistently make up at least 53% of the scholars and trainees, regardless of fiscal year. Female representation peaked for KL2 scholars in FY 2017 at 60% and for the TL1 trainees in FY 2018 at 59%. Consistently between FY 2011 and FY 2020, female representation in the KL2 Program was greater than in the Mentored Research Career Awardee portfolio that peaked at 52% in FY 2015 and FY 2018. Gender parity in the Mentored Research Career awardees took place in FY 2015. Gender parity existed in both the TL1 program and the NRSA training grants as early as FY 2011.

### Racial/Ethnic Distribution of CTSA Program Scholars and Trainees

The percentage distribution of KL2 scholars and TL1 trainees by race and ethnicity is found in Table 4. These percentages were compared with scholars and trainees appointed on NIH-wide Institutional K awards and Institutional T awards. On average, between FY 2011 and FY 2013, the KL2 scholar population was comparable (roughly 60% non-Hispanic White) to the NIH-wide Institutional K award population (roughly 58% non-Hispanic White). Between FY 2014 and FY 2019, the percent non-Hispanic White in both the KL2 scholar and the NIH-wide K scholar populations mirrored one another. Roughly 55% to 60% of the scholar population were non-Hispanic White. In FY 2019 and FY 2020, the KL2 scholar population was 55% and 53% non-Hispanic White, respectively, while the NIH-wide K scholar population was 57% and 56% non-Hispanic White in FY 2019 and FY 2020, respectively.

Shifting to racial and ethnic groups other than non-Hispanic Whites, Table 4 shows the percentage of KL2 scholar population who self-identify as non-Hispanic Asian or as a member of an underrepresented group (URG), as defined by Notice of NIH’s Interest in Diversity [13]. On average, between FY 2011 and FY 2020, roughly 20% of the KL2 scholars were non-Hispanic Asian. In contrast, there was a steady trend toward greater representation of URGs in the KL2 population during these fiscal years. Between FY 2011 and FY2014, roughly 12% of the KL2 population was from an URG. On average, between FY11 and FY20, 20% of the KL2 scholars were non-Hispanic Asian and 16% were from an URG; these averages somewhat mirror that of the NIH-wide K population with the CTSA Program KL2 scholar population becoming more diverse between FY17 and FY 20. During this period, the percent of scholars from URGs in the NIH-wide K population remained unchanged.

The diversity trend trajectory of the CTSA Program TL1 population has been less straight forward. The TL1 population has consistently been more diverse relative to the CTSA KL2 scholar population and the NIH-wide Institutional T population. Between FY 2011 and FY 2020, on average 55% of the TL1 trainees were non-Hispanic White compared to 59% of the NIH-wide Institutional T population. During this same period, the percentage of the non-Hispanic Asian population in the CTSA TL1 Program has decreased from roughly 21% to just below 15%. In contrast, the percent of members of URGs increased from 17% to 24%. The data suggest that the increase in the representation of URGs in the CTSA TL1 is a result of a decrease in appointment of non-Hispanic Asians. The story is slightly different in the NIH-wide Institutional Ts. The percent of non-Hispanic Asian trainees has been a constant at 16% to 17% from FY 2011 to FY 2020, while the percent of members from an URG has slowly, but steadily increased from 19% to 24%.

## Discussion

To our knowledge, this is the first report that describes the demographic characteristics of the CTSA Program hub PIs, the KL2 scholar, and the TL1 trainee populations. Analyses of workforce demographic reveal that although progress has been made, women and members of URGs remain persistently underrepresented in the CTSA hub and training components PIs. In contrast, in the career development and training programs, females have greater representation as scholars and trainees, and non-Whites are better represented in the KL2 and TL1 programs.

### Workforce Landscape

In FY 2011, females comprised roughly 18% of the CTSA hub and training components PIs; by FY 2020, this figure had grown to 34%. This is important and meaningful progress. Notably, the increased representation of females took place during a period when female representation also increased in the NIH RPG portfolio, in the NIH Center grantee population, and in academic medicine. The AAMC’s *2018–2019 The State of Women in Academic Medicine: Exploring Pathways to Equity Report* [17] documents the steady rise in the number of females in the academic medicine leadership structure. Among the administrative faculty leaders in academic medicine, by 2018, females comprised 52% of assistant deans; 47% of associate dean; 34% of senior associate/vice dean; and 18% of department chairs. The percent females in the CTSA Program hub leadership mirrors that of the NIH RPG portfolio and NIH Center Grants, but less so as medical school deans. Although the representation of females across the ranks of academic medicine continues to need improvement, there appears to be a rich pool of females who can progress into the CTSA Program hub leadership.

There is the recognition that the gains made by women remain fragile [17,18]. The COVID-19 pandemic has brought this situation to the forefront. Women have had career disruptions in terms of productivity, engagement, networking, and boundary control, just to name a few [18]. Efforts to recruit and retain women in science remain paramount.

In contrast, females are well-represented in the KL2 scholar and TL1 trainee populations, which is consistent with previous findings. According to Hechtman et al. [19] and the AAMC [17] gender parity exists among biomedical science doctoral degree holders and close to parity among medical school graduates. However, Valantine et al. [9] present compelling data highlighting female attrition across training and career stages. Although females make up more than half of biomedical science earlier in the pathway (58% as undergraduates and 53% as postgraduates), only 18% of full professors in the biomedical sciences are female [20]. A common assumption is that females are less successful in obtaining first-time grants and experience an accelerated attrition stemming from a lack of funding longevity relative to males. However, Hechtman et al. [19] dispel these assumptions. Specifically, they report that women had similar funding longevity as men after they received their first major NIH grant. The attrition of the representation of female in leadership is the result of other factors. Some reasons include, but are not limited to, implicit bias towards hiring men; [21] lower starting packages and salary for women that tends to have long-term professional implications; [21] and the lack of female role models at the leadership level [22]. Cultivating and nourishing gender diversity will ensure a healthy pathway for a long-term and sustained female representation within the CTSA Program hub leadership.

Unlike gender diversity, the representation of historically URGs in the CTSA Program hub and training components PIs highlights opportunities for improvement. At least four-fifths of the CTSA hub and training components PIs has been, and continues to be, non-Hispanic White. The lack of diversity in academic medicine is not new. For instance, the AAMC [23], documents that members of URGs are less likely to be promoted to senior ranks than their non-Hispanic White counterparts. Their data also show that less than 10% of the full-time medical school faculty are from URGs [23]. This has implication for leadership progression. Yu et al. [24] found that 87.8% of department chairs and 91.3% of Deans in academic medicine from 1997 to 2009 were non-Hispanic White. While Kaplan et al. [25] argue that grant funding and publications are essential for obtaining leadership positions. The culture in academic medicine is cited as contributing to the lack of diversity [25]. As with gender bias, academic medicine is not free of race and ethnicity-based implicit bias and is not free of other barriers that contribute to attrition of individuals from URGs [26].

While the lack of diversity in the KL2 scholar population is not as pronounced, there exists significant opportunities for improvement. The 2020 US Census estimates that 33% of the United States population report being from a group that is underrepresented in the biomedical workforce [27]. The KL2 scholars are those closest in the career pathway to becoming leaders in CTS; lack of diversity among this group has implications for the leadership pathway, both short and long term [12]. The TL1 trainee population tends to be more diverse. These findings are consistent with previous findings suggesting that members of URGs tend to be “better” represented earlier in the educational pathway. However, there is a striking loss of individuals along the entire pathway [9]. This loss means that although non-Hispanic White and Asian males make up 35% of all undergraduate in science, technology, engineering, and mathematics (STEM) fields, they represent 43% of all biomedical doctorates, and more than 82% of all full professorships [9].

Valantine et al. [9] contend that there is a loss of members of URGs in the pathway rather than a lack of a viable “pool.” In 2014, the National Academy of Science reported that in the last 25 years the number of postdoctoral researchers had doubled in the USA. This represented 60,000 to 100,000 postdoctoral researchers – a large fraction of these holding degrees in the biomedical sciences. The percentage of non-White undergraduates between 1996 and 2016 grew from 29.6% to 45.2%, and the percentage of non-White graduate students grew from 20.8% to 32% [28]. These data suggest that a racial and ethnically diverse candidate pool exists at the predoctoral and postdoctoral levels.

## Conclusion

Diverse perspectives enhance science. This report addresses the paucity of information about the self-reported demographic characteristics of the CTSA Program hub leadership of the KL2 scholar and TL1 trainee populations. The data have yielded several important findings and implications. First, there has been an increase in the representation of females in the CTSA Program hub leadership. Females are moderately represented in the CTSA Program hub leadership and their representation requires improvement. Gender parity has been achieved in the KL2 scholar and TL1 trainee populations, however. Second, the CTSA Program hub community needs to enhance its commitment to increase racial and ethnic diversity. Although the representation of females and racial and ethnic groups in the CTSA Program resemble that of NIH-wide RPG, NIH Center PI population, NIH workforce or AAMC data, it is not enough to be equal to or aspire to maintain the status quo. Research excellence and productivity come with greater diversity in the academic medicine workforce [10]. Building and leveraging a diverse and robust CTS enterprise can be a source of motivation for the CTSA Program community, as it is for NIH and NCATS. To achieve this goal, NCATS has taken steps to encourage inclusivity by enhancing the DEI language in the CTSA Funding Opportunity Announcements [29]. Similarly, the CTSA Program hub community has shown its commitment to DEI through Cohorts for Change [30], the Fall 2020 Meeting, and a taskforce to tackle this important topic. The CTSA Program community can set an example and help advance DEI within academic medicine and within the greater the biomedical research enterprise so that the faces and gender of the future CTS workforce looks more like the communities they serve.

## Footnotes

1
eRA Commons or Electronic Records Administration is an online interface where grant applicants, grantees, and federal staff at NIH and grantor agencies can access and share administrative information relating to research grants.
